# Recent Progress in Lipid Nanoparticles for Cancer Theranostics: Opportunity and Challenges

**DOI:** 10.3390/pharmaceutics13060840

**Published:** 2021-06-07

**Authors:** Sarah I. Bukhari, Syed Sarim Imam, Mohammad Zaki Ahmad, Parameswara Rao Vuddanda, Sultan Alshehri, Wael A. Mahdi, Javed Ahmad

**Affiliations:** 1 Department of Pharmaceutics, College of Pharmacy, King Saud University, Riyadh 11451, Saudi Arabia; sbukhari@ksu.edu.sa (S.I.B.); simam@ksu.edu.sa (S.S.I.); salshehri1@ksu.edu.sa (S.A.); wmahdi@ksu.edu.sa (W.A.M.); 2 Department of Pharmaceutics, College of Pharmacy, Najran University, Najran 11001, Saudi Arabia; mzahmad@nu.edu.sa; 3 Research Centre for Topical Drug Delivery and Toxicology (TDDT), University of Hertfordshire, Hertfordshire AL10 9AB, UK; p.r.vuddanda@herts.ac.uk; 4 Department of Pharmaceutical Sciences, College of Pharmacy, Almaarefa University, Riyadh 11597, Saudi Arabia

**Keywords:** cancer, multi-drug resistance, enhanced permeation and retention effect, nanotherapeutics, cancer theranostic, clinical translation

## Abstract

Cancer is one of the major leading causes of mortality in the world. The implication of nanotherapeutics in cancer has garnered splendid attention owing to their capability to efficiently address various difficulties associated with conventional drug delivery systems such as non-specific biodistribution, poor efficacy, and the possibility of occurrence of multi-drug resistance. Amongst a plethora of nanocarriers for drugs, this review emphasized lipidic nanocarrier systems for delivering anticancer therapeutics because of their biocompatibility, safety, high drug loading and capability to simultaneously carrying imaging agent and ligands as well. Furthermore, to date, the lack of interaction between diagnosis and treatment has hampered the efforts of the nanotherapeutic approach alone to deal with cancer effectively. Therefore, a novel paradigm with concomitant imaging (with contrasting agents), targeting (with biomarkers), and anticancer agent being delivered in one lipidic nanocarrier system (as cancer theranostics) seems to be very promising in overcoming various hurdles in effective cancer treatment. The major obstacles that are supposed to be addressed by employing lipidic theranostic nanomedicine include nanomedicine reach to tumor cells, drug internalization in cancer cells for therapeutic intervention, off-site drug distribution, and uptake via the host immune system. A comprehensive account of recent research updates in the field of lipidic nanocarrier loaded with therapeutic and diagnostic agents is covered in the present article. Nevertheless, there are notable hurdles in the clinical translation of the lipidic theranostic nanomedicines, which are also highlighted in the present review along with plausible countermeasures.

## 1. Introduction

At present, cancer is one of the leading causes of mortality worldwide. As per the demographic provided by the world health organization (WHO), cancer is accountable for approximately 10 million deaths in 2020 [1,2,3,4]. The emerging area of nanotechnology has proved very promising in cancer therapeutics [5]. Despite tremendous efforts in the research area of carcinomas alleviation through nanomedicines, there are a very few approved nanomedicines such as Doxil^®^, Myocet^®^, Abraxane^®^, Depocyt^®^, Genexol^®^ [5,6]. The FDA-approved Doxil was thought to be a revolutionary lipidic nanomedicine when it was successfully developed. However, it was demonstrated in a clinical study that it exhibited poor therapeutic efficacy [7,8]. Harrington and colleagues conducted a study in which they monitored tumor uptake of ^111^In-labelled poly(ethylene glycol) coated (PEGylated) liposomes in 17 patients with locally advanced cancers [9]. In this study, they were able to successfully demonstrate the major reason for the therapeutic failure of Doxil [9]. The study outcome revealed a very important and highly ignored aspect of in vivo fate of nanomedicines, targeting different forms of carcinomas. The study has confirmed that the accumulation of ^111^In-liposomes did take place in tumor restricted areas, but the concentration of nanoformulation that was retained in the tumor cells varied largely across different patients and tumor types [9,10]. In cancer patients, it has been established that enhanced permeation and retention effect (EPR) in which tumor vasculature becomes impaired and lymphatic drainage becomes deficient, felicitates drug accumulation in tumor cells, but that concept cannot be generalized after such findings [9,10]. Several factors affect nanomedicine accumulation and retention in tumor cells that need substantial consideration, such as tumor heterogeneity that causes diversified uptake of nanomedicines [11,12]. Tumor heterogeneity could be attributed to the distinctive cellular morphology, expression of efflux or influx transporters such as P-glycoprotein, presence of receptors, gene expression, metabolism, proliferation, and metastatic potential.

**Figure 1:**
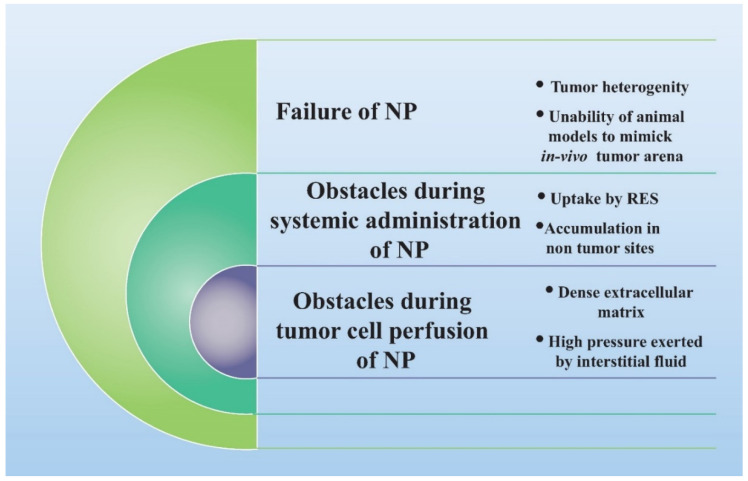
Barriers to efficacious in vivo performance of nanomedicines.

Besides, there is considerable constraint at different levels of nanomedicine administration (as shown in Figure 1) journey in vivo that governs the overall therapeutic efficacy of nanomedicines. When nanomedicine is administered systemically, then it encounters different physical, chemical, and physiological barriers that hinder its reach to tumor cells. After getting across these barriers, there are significant hindrances at the tumor microenvironment level that severely affect drug accumulation [11,12,13]. These hindrances include abnormal structure and highly variable density of tumor vasculature that greatly interfere with the optimal diffusion of nanomedicines. In addition, the pressure exerted by interstitial fluid and tightly packed tumor cells is very high, which avert the diffusion of nanomedicines across the tumor milieu. Moreover, a highly dense extracellular matrix greatly restricts nanoparticle extravasation and interstitial diffusion (Figure 1) [11,12,13].

Therefore it was realized that there is a major lacuna in our knowledge of the pathophysiological complexities and heterogeneity of tumor sites that affect the therapeutic efficacy of nanomedicines. Those patients are not even identified who are likely to benefit most from given nanomedicine-based chemotherapy [14].

**Figure 2:**
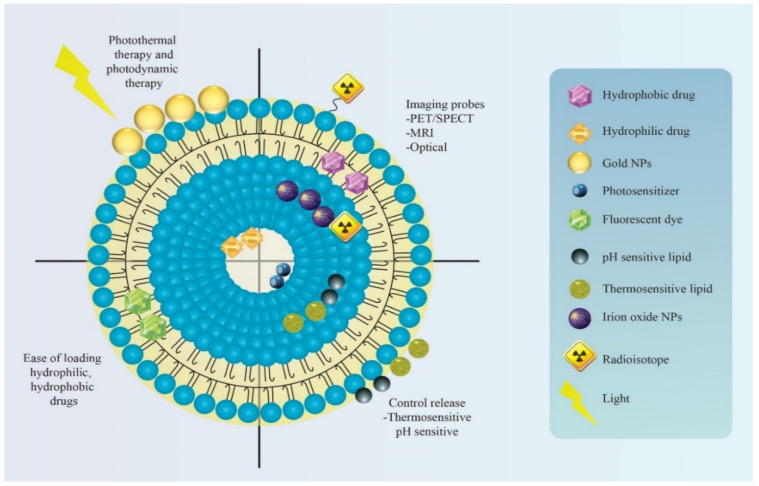
Brief depiction of how lipidic theranostic can help in cancer alleviation via multi-functionalized aspects such as coupling of the imaging probe, surface with gold nanoparticles for photothermal therapy.

Then next, the in vivo nanomedicine behavior knowledge is restricted to animal data and the animal models used do not mimic the actual in vivo conditions [15,16,17,18,19,20]. Usually, the NPs targeted for solid tumors after systemic administration are accumulated in the tumor through the EPR effect. Nevertheless, several crucial aspects related to EPR interpretation have been greatly overlooked, such as the influence of nanomedicine–protein interaction, blood circulation, tumor tissue penetration, and tumor cell internalization. Furthermore, all these biological processes are greatly affected by nanomedicine properties (for example, size, geometry, surface features) thus there are so many factors governing EPR effects driven in vivo nanomedicine behavior that cannot be predicted from animal data for humans. To date, there is not a single model that can completely replicate the entire facets of human malignancy [15,16,17]. This issue can be addressed if diagnosis and therapy can be combined as one approach in developing lipidic nanomedicines targeting cancers (Figure 2).

A cancer diagnosis has a very significant role in the context of precision of chemotherapeutic medication. Diagnosis specifically implicates recognizing the presence of a tumor in the body and evaluating its extent to identify if it is at its early developmental stage or re-occurrence case [1,2]. Importantly, identification of precancerous lesions could result in a successful intervention of cancer and its complete alleviation. Early diagnosis is certainly life-saving in cancer treatment. Once the existence of cancer is confirmed, diagnostic tools are implicated in identifying specific molecular abnormalities in tumors that govern the medications to be provided accordingly. Nowadays, with advances in biomedical technologies, novel diagnostic approaches are being investigated that will enable the identification of cancerous and precancerous cells at the molecular level and provide information about their pathophysiology. The combination of diagnostics with therapeutics makes it easy to know the progress of treatment and the real-time state of cancer while receiving therapy. Moreover, imaging agents/drug trackers can help greatly to know the in vivo fate or traveling of a drug in systemic circulation or at the tumor and can also determine the kinetics aspect of anticancer drug/drug loaded formulation [20,21]. That is why the use of imaging biomarkers such as radioactive substances taken up by tumors and visualized through diagnostic modalities such as Computed Tomography (CT), Magnetic resonance imaging (MRI), positron emission tomography (PET), and single-photon emission computed tomography (SPECT) is attaining a lot of attention in oncology these days. Taking into consideration the phenomenon of tumor metastases on the way to cancer mortality, the combinatorial approach of diagnosis and treatment that is theranostic will be of substantial importance for the assessment of EPR and nanomedicine penetration. The clinical translation of anticancer nanomedicines could see a breakthrough outcome via the introduction of a theranostic approach that can intently trace the in vivo fate of drugs and assess the progression of alleviation of human tumors via encapsulated nanomedicines [18,19,20,21].

Therefore, the diagnosis in combination with therapy is quintessential for embarking on a level of cancer treatment that could offer highly efficacious clinical outcomes. In this review, an insight of a combination of therapy and diagnosis, which is called theranostic, is provided, covering the brighter prospects and the challenges accompanied with it. The lipidic nanomedicine-based theranostic is also the highlight of this present review. A comprehensive account of different research updates in the field of lipidic nanocarrier loaded with diagnostic agents is envisaged. Nevertheless, there are notable hurdles in the clinical translation of the lipidic theranostic nanomedicines, which are also discussed in the upcoming section of the present review.

## 2. Significance of Lipid-Based Theranostic Nanoparticles in Cancer Therapy

Early detection of carcinomas is of great pertinence for their successful alleviation. Firstly the diagnosis of the type of tumor, its metastatic state, and the patient history need to be identified to initiate the appropriate therapy. Here theranostic plays a crucial role. Once correctly and profoundly diagnosed, then the nanomedicine-based therapy could be started relevantly. However, the monitoring of the in vivo fate of nanomedicine is a vital aspect of assessing the progression and efficacy of cancer therapy. That is why co-encapsulation of imaging agents and drugs in a single nanocarrier system could contribute significantly in assessing the progression of treatment and exact and precise state of the response of cancer towards provided therapy [21,22,23].

**Figure 3:**
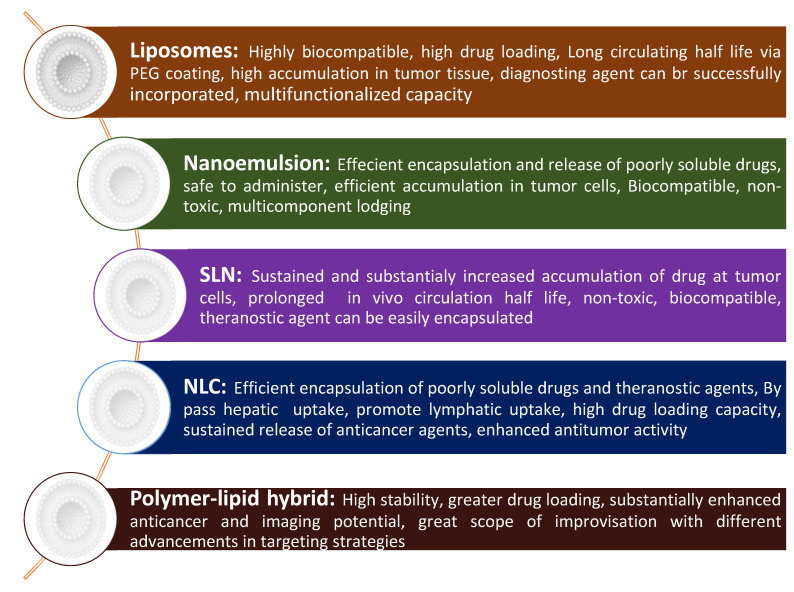
Potential advantages of different types of lipidic formulations in cancer theranostic.

**Figure 4:**
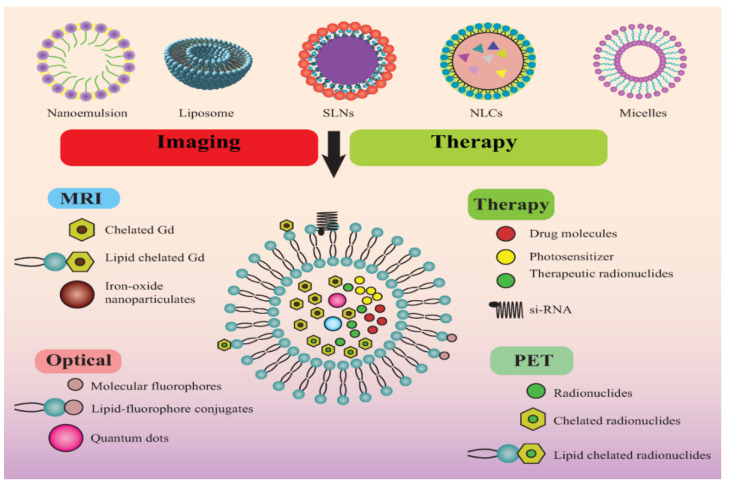
Different types of lipid nanoparticles *viz.* nanoemulsion, liposome, solid lipid nanoparticle (SLN), nanostructured lipid carrier (NLC), and micelles with significant utility in cancer imaging and therapy.

Theranostic nanomedicine could be prepared in multiple manners. The lipid nanocarriers have been proven superiority over polymeric and inorganic nanoparticles in terms of biocompatibility, safety, and biodegradability besides other beneficial considerations from a cancer theranostic perspective (as summarized in Figure 3) [7,8,9]. Therefore, in this review, we are concerned with lipidic theranostic for cancer, and we will be focusing on the vesicular or micellar lipidic structures. Different shapes and structures can be formulated depending on the type of lipidic nanocarrier chosen (as shown in Figure 4). In nanoemulsions, the theranostic agent and drug are entrapped in oil globules targeted for specific tumor sites. In liposomes, imaging agents can be encapsulated with drugs either in an aqueous core or bilayer lipidic shell. In solid lipid nanostructure (SLN), the imaging agent is intercalated in the solidified lipid matrix. Whereas, in nanostructure lipid carrier (NLC), the drug and the imaging agent are dispersed in the imperfections of oil and solid lipid hybrid matrix. However, optimizing lipid theranostic nanomedicines with balanced size, shape, polydispersity index, surface charge, and stability, is a challenging task in itself that we will discuss in detail in later sections.

The imaging agent that is incorporated with the drug in lipidic vesicles must possess great compatibility with conventional diagnostic techniques such as X-ray, ultrasound (US), CT, MRI, PET, or SPECT [24]. The contrasting agents used in theranostic usually include metals or inorganic agents (such as iron oxide) as they exhibit free electrons whose excitatory phase can be used as imaging modalities (MRI). Different semiconductor-based nanoparticulate systems (such as quantum dots) of colloidal dimension are also being extensively employed in in vivo diagnostics [25]. Fluorescent silicon nanoparticles are also employed as an imaging agent for foreseeing in vivo prospects [26].

MRI is one of the most commonly used diagnostic modalities, which depends upon mobile protons of water molecules for detecting signals. The MRI images can be perceived via proton concentration in body tissues or else via their longitudinal and transversal relaxation times, T1 and T2, respectively. A plethora of contrast agents are being used for MRI. Amongst them, fluorine-containing contrast agents have garnered splendid attention. Owing to its excellent magnetic property, 100% natural abundance, one-half spin, sensitivity, and gyromagnetic ratio comparable to a proton, fluorine has become the contrast agent of choice for MRI. Furthermore, with ^19^F in vivo tracing and imaging can be accomplished without any background signals created by endogenous fluorine. Nonetheless, there has been a limitation with fluorine as its large concentration (10–50 millimolar) is needed for adequate signal intensity in comparison to other contrast agents [27].

The promising combination of imaging along with nanomedicine-based therapy has the extremely worthy potential of overcoming the pathophysiological hurdles that undermine the efficiency of cancer therapy. The simultaneous examination of nanomedicine reach to tumor cells, amount of drug release, off-site tumor drug distribution, and uptake via host immune system are those aspects, which can tremendously affect the therapeutic outcome.

Until recently, the lack of interaction between diagnosis and treatment has hampered efforts to deal with cancer effectively. This new paradigm with simultaneous multifunctionality of imaging (with contrasting agents), targeting (with biomarkers), and delivering chemotherapeutic agents in one lipidic nanocarrier system seems to be very promising in overcoming various hurdles in effective cancer treatment.

## 3. Different Types of Lipid Nanoparticles for Cancer Theranostics: An Update of Recent Studies

Biocompatibility and safety are the major reasons for choosing a lipidic nanocarrier system as cancer theranostic in this review. Certainly, prolonged in vivo circulation half-life, high encapsulation capacities for drug and imaging agents, substantial accumulation at tumor sites, and improvisation susceptibility for multiple functioning are the other significant advantages with lipidic nanocarrier system [28,29,30]. Lipidic nanocarriers have their own sets of advantages, which give them an edge over other nanoformulations. Recent research studies demonstrated lipidic theranostic nanomedicines to be a promising and potential approach for raising the efficacy of cancer treatment to a hallmark level, as discussed hereunder. The different lipidic nanocarriers include liposomes, nanoemulsions, SLN, NLC, and lipid nanocapsules. However, advanced lipidic nanocarriers such as SLN and NLC are still unexplored for cancer theranostic as there are very few studies conducted thus far.

### 3.1. Nanoemulsion

**Figure 5:**
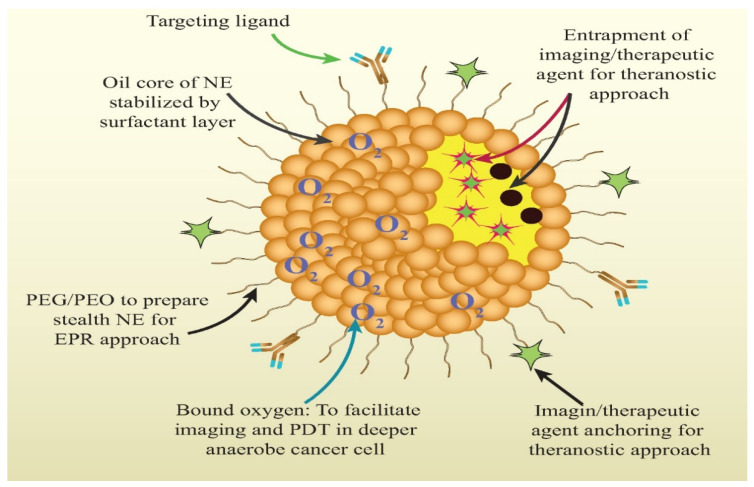
Brief depiction of how lipidic theranostic can help in cancer alleviation via multi-functionalized aspects such as coupling of the surface with targeting ligand; entrapped theranostic agent, PEG coating on the surface for evading systemic clearance, bound oxygen for enhancing photothermal therapy, surface anchored contrasting agent.

Nanoemulsions have gained huge attention for the efficient delivery of lipophilic anticancer drugs. The nanometric size, large surface area, thermodynamic stability, high drug loading capacity, easy scalability, biocompatibility, favorable drug release profile are the characteristic attributes of nanoemulsion that make it worthy of profound exploration in chemotherapeutics [31,32]. The nanoemulsions, mostly oil-in-water types, are optically transparent colloidal dispersion constituted of oil and water, wherein surfactants and co-surfactants create a stable coating over the dispersed droplets to form a physicochemically stable nanoformulation [32]. The excipients of nanoemulsion are generally recognized as safe (GRAS) grade making the formulation highly safe to administer with improved bioavailability and therapeutic efficacy. Different strategies have been reported of late, wherein contrast and chemotherapeutic agents are entrapped in nanoemulsion for selectively targeting tumor microenvironment (TME) for both diagnostic and therapeutic drive [33,34,35,36,37]. Nonetheless, there are many obstacles in their journey from animal models to afflicted cancer patients, including firstly physicochemical stability of theranostic nanoemulsion, subsequently, its in vivo fate, then degradation, and clearance from the system, along with long-term stability and toxicities concerns. Various attempts have been made thus far to surmount these hurdles and to entrap chemotherapeutic agents and diagnostic agents in nanoemulsion, particularly from the perspective of developing an efficient cancer theranostic agent that is highly capable of clinical translation (Figure 5).

The major challenge associated with developing lipid theranostic nanomedicine, which is also the major limitation in their clinical translation, is that the incorporation of several components in one nanocarrier [38]. This integration of numerous constituents in nanodispersion causes polydispersity, heterogeneity, and also difficulty in scalability issues [38]. In an attempt to address these issues, Zhang and coworkers 2020, developed fluorinated nanoemulsions with remarkably improved fluorescence imaging signals for selective and sensitive tumor detection [38]. Their theranostic approach was highly capable of selectively recognizing the specific type of tumor (integrin αvβ3 overexpressed cancer cells), potential tracing of in vivo fate of nanoemulsion, and offering highly efficient photodynamic therapy. The meticulous approach averted many ingrained concerns with conventional nanomedicine, including polydisperse polymers, heterogeneous constituents, and complex formulation. Importantly, this approach imparted multiple functional aspects to the nanoparticles with tumor-targeting accompanied with quantitative and sensitive multimodal imaging (FL, ^19^F MRI, ^129^Xe hyper-CEST MRI), and PDT with a high therapeutic index [38]. Huang and associates 2019, designed an integrated system of multimodal imaging signals and PDT function into a poly(ethylene glycol)-boron dipyrromethene amphiphile (PEG-F54-BODIPY) with 54 fluorine-19 (^19^F), as an “all-in-one” nanocarrier [39]. This novel amphiphile acquired various unique and desirable attributes that make it potential cancer theranostic agents. The developed nanoemulsion was having distinctive structure-based fluorescent, photoacoustic, and magnetic resonance properties and prolonged tumor retention time for repeated PDT treatment, and great biocompatibility. The study outcomes revealed in the melanoma cancer xenograft model, developed nanoemulsion can be efficiently used as an activatable nanoprobe with improved sensitivity of multimodal imaging for tumor recognition [39]. One more very interesting attempt was made by Fernandes and Kolios 2019, to increase the selectivity and targeting of nanomedicine for substantially afflicting cancer cells without causing any harm to nearby cells and off-tumor areas [40]. The fabricated perfluorohexane nanoemulsions possess the ability to selectively target cancer cells as these nanoparticles carry therapeutic agents, which have slow release rates and become vaporized into perfluorohexane bubbles without any rise in temperatures that could affect normal cellular function. Their ability to use higher concentrations of theranostic agents could be of significant advantage in improving therapeutic efficacy and imaging ability in clinical applications. In this study, the ability of nanoemulsions to cart therapeutic agents, doxorubicin, and paclitaxel, specifically for the treatment of breast cancer, was investigated. The study outcome revealed that perfluorohexane nanoemulsion could be efficiently internalized in cancer cells and could cause significant cell death. The developed nanoemulsion with concurrent laser excitation capability exhibited tremendous potential for destroying all tumor cells and emerging as a competent theranostic agent for averting the growth of cancer cells [40]. Furthermore, Zheng and associates, 2019 fabricated a novel nanoemulsion with a porphyrin shell (NewPS), and it was the simplest yet multifunctional nanoemulsion system developed thus far [41]. The porphyrin salt shell permitted the encapsulation and stabilization of the oil core, yielding a monodisperse, spherical nanostructure with excellent colloidal stability. The inherent multimodality of porphyrins enabled the multifunctionality of NewPS for imaging and phototherapy. Moreover, the oily core felicitates the efficient loading of hydrophobic anticancer molecules. The study established formable and intelligible, surfactant-free nanoplatforms for theranostic cancer applications. This novel two-component NewPS served as an innovative avenue for multimodal cancer imaging, phototherapy, and image-guided drug delivery [41].

In light of such studies, it is anticipated that nanoemulsions-based theranostic could offer promising opportunities in cancer treatment.

### 3.2. Liposomes

Liposomes stand tall in the crowd of conventional lipidic nanocarrier systems owing to their inimitable characteristic attributes. Their unique structure comprised of unilamellar lipid bilayers that nest an aqueous core offers great flexibility of easy incorporation of multicomponent. It also provides an option for both hydrophilic and lipophilic chemotherapeutic drugs and contrast agents. Besides biocompatibility, safety, biodegradability aspects, liposomes also offer an enormous scope of surface improvisation for selectively targeting tumor cells [42]. Recent literature highlights that liposomes have been amongst the topmost area of active research of cancer theranostics. At present, they are being largely investigated for incorporating and targeting cancer via contrast agent such as ^64^Cu [43] and ^14^C isotopes [44], QDs [45], gadolinium (Gd)-based contrast agents [46], super paramagnetic iron oxide particles (SPIONs) [47], and fluorescent probes [46,48].

The upcoming section will throw light upon the latest research studies that have confirmed the substantial potential of liposomes as cancer theranostic that could be successfully taken to clinics.

Prasad and associates 2021; fabricated liposomal nanotheranostics in which gold nanoparticles (AuNPs) and emissive graphene quantum dots (GQDs) were encapsulated along with a chemotherapeutic agent [49]. The surface of the liposome was functionalized with folic acid for targeted delivery. The prepared targeted liposomal theranostic demonstrated site-specific tumor diagnosis and photo-triggered tumor extirpation. The study outcome confirmed specific and enhanced cellular uptake, prolonged internalization in tumors, and substantial contrasting and therapeutic efficacy of nanotheranostic liposomes with dual imaging probes. The synergistic effect of anticancer drugs and photothermal effect exhibited superior tumor impedance, in contrast, to stand-alone therapy. Moreover, these multicomponent loaded liposomes have good colloidal stability, biocompatibility, and biodegradability as demonstrated in in vivo imaging. Therefore the developed nano hybrid liposome nanotheranostic served as a safe and tremendously potential platform for multifunctional tumor diagnosis and targeted therapy [49].

Furthermore, Karpuz and associates 2020, investigated the in vivo therapeutic prospect and contrasting efficacy of paclitaxel and vinorelbine loaded, Tc-99m radiolabeled, folate targeted nanosized liposomes [50]. The study outcomes demonstrated targeted delivery of chemotherapeutic agents, which got efficiently lodged in tumor vasculature and resided there for a prolonged time, causing substantial reduction of tumor growth. The in vivo images confirmed mitigated off-site accumulation and toxic effect of liposome theranostic nanomedicines [50].

In another interesting research study, a very serious issue of brain metastasis (BM) and tyrosine kinase inhibitor (TKI) resistance that are the two major challenges in non-small cell lung cancer (NSCLC) treatment were addressed [51]. Yin and associates designed a dual-targeting liposomal system for co-delivery of gefitinib and simvastatin to treat BM of NSCLC. The study confirmed via fluorescence imaging that dual-targeted liposome could efficiently cross the blood–brain barrier and is highly capable of reversing drug resistance. Therefore, the developed liposomal formulation represents a potential strategy for treating advanced NSCLC patients with BM [51].

Bush and coworkers 2020 also came up with an interesting concept of acoustic cluster therapy (ACT) that constitutes of co-administration of a formulation containing microbubble constellations, along with regular anticancer drug and local US insonation of the targeted tumor tissue. The microbubble cluster efficiently resided in the tumor’s microvasculature [52]. The therapeutic efficacy of ACT with liposomal doxorubicin for the treatment of triple-negative breast cancer using orthotopic human tumor xenografts in athymic mice was assessed. The study outcome established substantial increase in the therapeutic efficacy of Doxil^®^ when combined with ACT [52]. Another crucial study that encourages the concept of application of liposome in cancer theranostic was conducted by Prabhakar and Banerjee 2019 [53]. They formulated submicron-sized (528.7 ± 31.7 nm) nanobubble-paclitaxel liposome (NB-PTXLp) complexes for ultrasound imaging and ultrasound responsive drug delivery in cancer cells. The concept resulted in more than 300-fold higher anticancer activity of NB-PTXLps in the presence of ultrasound in MiaPaCa-2, Panc-1, MDA-MB-231, and AW-8507 cell lines, in contrast to commercial formulation Abraxane^®^. Therefore, the novel NB-PTXLps served to be a promising and triflingly invasive theranostic scaffold for cancer therapy in the forthcoming scenario [53].

The research studies discussed in this section undoubtedly unveiled enormous opportunities to facilitate the targeted chemotherapeutic delivery with concomitant in vivo imaging utilizing liposomes. Further research is envisaged to take these studies to clinical trials.

### 3.3. Solid Lipid Nanoparticles (SLN)

The second-generation lipid nanocarrier includes SLN, which are spherical colloidal nanoparticles with a solid lipid core comprised of waxes, triglycerides, fatty acids, and are stabilized by surfactants. Their size usually falls within the 50–100 nm range and is exclusively known for their biocompatibility, higher susceptibility of lymphatic uptake, and sustained drug release [54,55]. In cancer, alleviation chemotherapeutics loaded SLN is very promising [54,55,56]. Nonetheless, they are capable of efficiently carrying contrast agents along with anticancer drugs and provide simultaneous treatment and diagnosis, as evident in outcomes of recent research studies. Kuang and coworkers have demonstrated in their study that solid lipid nanoparticles (SLNs) conjugated with c(RGDyK) were designed as efficient carriers to improve the targeted delivery of IR-780 to the tumors [57]. The multifunctional cRGD-IR-780 SLN significantly improved the tumor-specific targeting, efficacy of photothermal therapy along with spontaneous imaging of in vivo journey of SLN incorporated IR-780 iodide nanomedicine [57].

SLNs have been investigated for integrating many contrasting agents, particularly superparamagnetic iron oxide [58], technetium-99 (^99^mTc), ^64^Cu [59], and quantum dots [60,61]. Very recently, a research study came up with SLN cancer theranostic wherein SLN was encapsulated with QD as a contrast agent [54,62]. The chemotherapeutic agent was the combination of Paclitaxel and siRNA. The solid lipid matrix was interspersed with paclitaxel and QD whereas siRNA, which was anionic, was electrostatically linked on the outer cationic surface. The combination of dual therapeutic agent paclitaxel and siRNA loaded in SLN got efficiently accumulated in lung carcinoma and exhibited synergistic anticancer activity. Importantly QD fluorescence in SLN made it possible to track higher in vivo cellular uptake of SLN on-site and mitigated uptake at off-site cancer cells. This study confirmed the potential theranostic applicability of SLN as a nanocarrier [54,62].

In another interesting research study, Morel and associates have revealed the successful integration of gadolinium (Gd) (III) complexes in SLN that would be contributing as an efficient and favorable oral contrast agent for MRI [63]. The percentage of Gd (III) complex entrapped in SLN was substantially higher as confirmed by average longitudinal relaxivity for Gd (III) complex in SLN system and pure water (25 °C, pH 7, 20 MHz) [63].

Another research study conducted by Andreozzi and associates is clear evidence of the multifunctional theranostic ability of SLN [59]. They radiolabelled SLN with ^64^Cu and assessed its bio-distribution by in vivo quantitative assessment, PET imaging, and ex vivo gamma counting. The study outcomes validated the simultaneous in vivo imaging and tumor ablation potential SLN theranostic, which is also safe, biocompatible, and biodegradable [59].

The research studies conducted thus far are very limited in numbers, and several other important aspects of SLN also need profound exploration from a cancer theranostic perspective, such as stimulation of lymphatic absorption by SLN. Whatever literature we have discussed can corroborate the efficient in vivo imaging and drug delivery utilizing SLNs along with the safe theranostic application, biocompatibility, and biodegradability, of developed nanomedicine. The results established that the SLN theranostic nanoformulation developed is optimistic for hallmark contribution in the field of cancer treatment with simultaneous diagnosis.

### 3.4. Nanostructured Lipid Carriers (NLC)

NLC is a smart next-generation nanocarrier with a unique hybrid matrix of solid and liquid lipids stabilized by surfactants. The imperfect crystalline or amorphous structure possesses an enormous potential of high drug loading and improvised drug release. Latest research studies indicate the promising role of NLC in cancer theranostic. Of late, Li and coworkers successfully developed a multifunctional nanocarrier of Coumarin 6 fluorescent dye and IR 780 encapsulated CXCR4-targeted NLCs for breast cancer alleviation also employing photodynamic therapy [64]. The developed system proved to be highly efficient in debilitating tumor progression and metastasis and concurrently allowing imaging [64].

Olerile and coworkers prepared a NLC loaded with QD and paclitaxel that was highly capable of monitoring and tracking tumor growth and simultaneously impeding tumor cells in the murine tumor model of hepatocellular carcinoma [65]. Researchers confirmed the great cancer theranostic potential of NLC as it was efficiently enabling targeted delivery with concomitant in vivo imaging [65].

Another very promising approach in the area of cancer theranostic utilizing NLC was reported. Camptothecin encapsulated-NLC was formulated conjoined with QD and fluorescent imaging probes that were capable of detecting the lodging, internalization, cytotoxicity, and biodistribution of NLC nanomedicine [66]. The study outcome established that NLC coordinated with QDs and an anticancer drug offered efficient tumor imaging and drug delivery and such accomplishment with a novel nanocarrier system was remarkable and worth mentioning here [66].

An interesting attempt was made to deliver paclitaxel-loaded NLC with folic acid dispersed hybrid lipid matrix [67]. The paclitaxel-loaded NLC was radiolabeled with ^99m^Tc(CO)^3+^. Due to the imperfection in a matrix structure, too many components were efficiently loaded in NLC as reflected by in vitro stability of developed formulation, which was up to the mark (>90%). Results indicated that ^99m^Tc(CO)^3+^-radiolabelled paclitaxel NLC was capable of identifying folate receptors overexpressed in tumor cells. The developed formulation was successfully localized at the specific targeted tumor areas without any off-site distribution and the uptake by RES on intravenous administration. The concurrent imaging efficiently depicted the in vivo fate of paclitaxel-loaded NLC that is very much desirable for an optimal therapeutic implication [67].

As the data concerning NLC theranostic for cancer is very restricted, there is a great need for a lot more investigation to be envisaged to explicitly explore multiple beneficial aspects of advanced lipid nanocarrier and pave the way for their successful clinical translation.

### 3.5. Lipid Nanocapsules (LNCs)

LNCs are also amongst the next-generation lipid nanocarrier systems with lipoprotein-like structures whose size falls within 1–100 nm. The structure of LNCs is a blend amidst polymeric nanoparticles and liposomes as they have an oily core with an exterior built of a tensioactive rigid membrane. LNCs are a novel lipid nanocarrier system and can be prepared via phase inversion of emulsions and organic solvent-free-based procedures [68].

Nevertheless, researchers consider LNCs as an optimistic platform for cancer theranostic as well. A very recent study further confirmed the promising outlook of LNCs in cancer theranostic. To selectively target tumor cells, QDs-based lipid nanocapsules (LNCs) encapsulated with celecoxib, and honokiol were designed and investigated. The study outcome revealed efficient accumulation and intracellular uptake of LNCs in tumor cells, and their internalization was progressively traceable via fluorescence restoration. The LNCs established highly improved and superior anticancer efficacy of LNCs against breast cancer cells. The developed system could be applied as a potential multifunctional nanotheransotic for imaging and targeted therapy of breast cancer [69].

### 3.6. Lipid-Based Micelles

Lipid-based micelles are the spherical structure of lipid molecules, in which they aligned themselves in aqueous solutions. This class of lipid nanocarrier system is unexplored to date, but it could also serve as a potential and promising therapeutic cum diagnostic nanomedicine for cancer treatment. In a study, Ma and coworkers developed a lipid-based micelle encapsulated with docetaxel (M-DOC) that possessed marked anticancer efficacy and mitigated toxicity in the xenograft breast cancer model [70]. The lipid-based micelles need to be duly explored for their cancer theranostic potential in the near future.

Table 1 enlists the different lipidic nanomedicine investigated for the theranostic applications in cancer and summarizes their theranostic outcomes in different experimental models.

## 4. Advancement in Lipid-Based Nanoparticles for Cancer Theranostics

### 4.1. Polymer-Lipid Hybrid System

Lipid polymer hybrid is next-generation lipid nanocarriers and has obtained its foundation from the amalgamation of liposomes and polymeric nanoparticles. They have a polymeric core enclosed by a lipid bilayer shell kind of structure. Many researchers claim it to be a very promising nanocarrier for anticancer drug delivery, however, its potential is not duly tapped and remains unexplored to date. In recent research, Huang and associates developed multifunctional tumor-targeted polymer-lipid hybrid nanoformulation, which was loaded with ultrasound contrast agents (glutathione (GSH)) and prodrug (Pt(IV)) for targeted delivery of theranostic nanomedicine against ovarian cancer. The nanosized formulation was composed of a perfluorohexane (PFH) liquid core, a hybrid lipid-polymer shell, and an active targeting ligand, which demonstrated improved cellular uptake. The study findings established Pt(IV) encapsulated lipid-polymer as a novel multimodality platform exhibiting excellent anticancer activity and substantially reduced toxicity and signifying a powerful theranostic nanomedicine for combating ovarian cancer [71]. The research outcome encourages further exploration of this class of lipidic nanocarriers to be envisaged for beneficial prospects in the field of cancer theranostic.

### 4.2. Endogenous High-Density Lipoprotein Derived Nanoparticles

Nanocarrier systems comprising endogenous high-density lipoprotein (HDL) could emerge as potential lipidic nanocarrier-centered cancer theranostic options owing to their non-immunogenicity, complete biodegradation, and infrequent reticuloendothelial system (RES) uptake [72,73,74]. HDL-like peptide- phospholipid nanovesicles (HPPS) imitate the structural and functional attributes of plasma-derived HDL [74,75,76,77]. He and coworkers recognized a TfR mAb (monoclonal antibody) tailored nanomedicines for improved tumor targeting. They demonstrated that drug entrapped HPPS based nanomedicines adapted with explicit ligands could muddle to receptors on the surface of tumor cells, resulting in the accretion of nanomedicines on the exterior surface of targeted cells [75,76,77,78,79,80,81,82,83,84,85,86,87,88,89,90]. It was concluded that developed HPPS based nanomedicine holds the potential to strengthen targeting to tumor cells and attains favored recognition by specific antibodies in a complex biological setting [91]. Such intricately woven novel research concept needs to be brought forth and encouraged thus that these kinds of potential research perceptions can be multiplied. Huge benefits can be extracted from such studies for accomplishing successful targeting of lipidic cancer theranostic nanomedicine.

Fayad and coworkers presented very interesting work on HDL-based multimodal nanotheranostic for targeting and imaging tumors [92]. The developed HDL nanoparticles got non-selectively accumulated and selectively binding to angiogenic active blood vessels. For targeting such angiogenic endothelial cells, HDL was reconstituted with gadolinium chelates and fluorescent dyes, and their surface was functionalized with αvβ3-integrin-specific RGD peptides. The incorporation of such peptides felicitated uptake of HDL-based nanoparticles by angiogenic endothelial cells as visualized in MRI after administration of developed nanoparticles in tumor-bearing mice. The study findings demonstrated the substantial possibility of redirecting HDL from their natural route towards tumor-ridden blood vessels along with successful imaging and tracing of an entire pathological process [92].

### 4.3. Hybrid Lipid-Inorganic Nanomaterials

Most recently, there has been a paramount focus shifted towards an exploration of hybrid lipid-inorganic nanomaterials, which combine and multiply the desirable attributes of both classes of nanocarriers, including lipidic nanocarriers and inorganic nanoparticles. The lipid nanocarrier system employed for such application includes liposomes, nanoemulsion, solid lipid nanoparticles, and lipoproteins. In contrast to singly functionalized counterparts, this hybrid multifunctional system offers many beneficial outcomes in cancer theranostic such as stimuli-triggered anticancer drug release, photothermal therapy, and bioimaging. The internalization of inorganic material inside the lipid nanocarrier governs their functional aspects as there are different spatial lodging based on the structure of lipidic assemblies. The inorganic material can reside in the surface coating of lipid nanocarriers as surface-bound nanomaterial, or it can be lodged in bilayer lipids lamellae in liposomes, and certainly, the inorganic material can also be internalized in the core structure of lipidic nanocarrier [93]. A plethora of investigational studies are reported in which gold (Au) was used as an inorganic nanomaterial for preparing hybrid lipid-inorganic nanoparticles for cancer theranostic. The considerably low toxicity, ease of improvising surface chemistry, tunable size and shape, and substantial electronic property make Au a metal of choice for preparing inorganic nanoparticles and their hybrid lipid nanoformulations that have great potential for bioimaging site-specific drug release, and photothermal cancer therapy [93]. Other potential inorganic materials that have been explored for preparing hybrid lipid-inorganic nanomaterial include silver and palladium nanoparticles. In addition, one of the most commonly used approaches include SPIONs, which are ideal contrast agent for MRI owing to their biocompatibility and distinctive magnetic properties [94]. Moreover, the potential of semiconducting nanoparticles (QDs) is becoming widely accredited in bioimaging as optical probes over conventional organic dyes [95].

The recent study findings focusing on hybrid lipid-inorganic nanomaterials are clear evidence of their potential in cancer theranostic. In a study, Kang and Ko have developed a hybrid lipid inorganic nanomaterial by efficiently incorporating docetaxel in Au nanoparticles and then encapsulating this Au nanoparticle in thermosensitive phospholipid lipid bilayer coating [96]. The outcome of the study established enhanced cellular uptake, internalization, and cytotoxicity of hybrid lipid inorganic nanoformulation in comparison to uncoated drug-loaded Au nanoparticles. The study findings strongly encourage the implication of drug-encapsulated lipid-coated anisotropic nanoparticles for amplifying therapeutic prospects of chemotherapy [96]. The recent research concluded the feasibility of breast cancer cell detection via conformance of the inorganic metal-nanoemulsion hybrid [97]. An improvisation was made in perfluorocarbon or QD nanoemulsions by incorporating N-hydroxysuccinimide modified phospholipids in the surfactant formulation, as this would enable conjugation of prepared hybrid QD nanoemulsion with the amine groups in antibodies. Such antibodies targeting growth factors are overexpressed in human breast cancer cells, which would be easily able to bind with nanoemulsions. The research study demonstrated the selective linking of hybrid nanoemulsion with its target breast cancer cell line [97]. Interestingly, low-density lipoprotein (LDL) encapsulating Au nanoparticles were explored for their biolabeling capability [98]. Administration of dodecanethiolcapped Au nanoparticles loaded LDL in mice with B16-F10 tumor resulted in selective uptake by tumor-associated macrophages that play a vital role in metastasis of tumor cells. The study findings confirmed the substantial potential of hybrid Au-LDL nanoformulation in in vivo tracking and treating of tumors without causing off-site damage [98]. In another study by Bao and coworkers, hybrid liposome nanoformulation wherein paclitaxel Au nanoparticles were embedded in its bilayer lipid lamellae demonstrated remarkably prolonged release rate and extended circulation time. The hybrid exhibited notable potential for enhancing the therapeutic efficacy of incorporated anticancer agents [99]. Mounting evidence has elucidated the potential of palladium nanoparticles as contrast agents for photothermal and anticancer therapy. Nevertheless, research works focused on hybrid lipid assemblies incorporating such palladium nanoparticles established excellent anticancer activity as well as site-specific uptake and internalization of a therapeutic agent through these hybrids [100,101]

Certainly, there are serious toxicity concerns with the use of inorganic nanomaterial. For example, oxidation of silver (Ag) to toxic Ag^+^ ion in biological milieu can cause toxic effects that must be controlled. Here hybrid lipid encapsulation could come to the rescue of such lethal transformation. By tuning the lipidic membrane composition, the release of Ag^+^ ions can be prevented. Moreover, studies have established that the physicochemical features, size, shape, surface, coating, surface area contribute significantly in dictating the hybrid lipid inorganic nanomaterial toxicity and its biological interactions [102]. Therefore, skillfully designed hybrid lipid inorganic nanomaterial exhibits the potential of overcoming the toxicity issue of incorporated metals.

### 4.4. Cancer Tumor Cell Targeting Theranostic Vector

At present, the active area of targeting circulating tumor cells (CTC) via theranostic vectors before the homing and progression of carcinomas is largely explored for improving cancer therapeutic intervention. The CTCs are those cells that are detached from primary solid tumors and traverse through blood and lymph to form secondary tumors [103]. Therefore, detection and targeting of CTC may result in early diagnosis and prevention of cancer and its metastasis. Determination of CTC concentration in blood could provide valuable information about the diseased state [103]. Therefore, estimating CTC concentration would contribute significantly in monitoring remission and relapse and assessing response to chemotherapy [103]. In a study by Bhattacharyya and associates, antibody-targeted Au nanoparticles were employed for CTC detection in breast cancer cell line T47D [103]. The study outcomes established Au nanoparticles as a promising approach for detecting and capturing CTC in a photoacoustic flowmeter. The study findings established an abundant scope of diagnosis of disease at an early stage in various solid tumors and its successful treatment. Such encouraging findings mark a new paradigm for cell-specific molecular analysis for individualized cancer therapy via capturing of CTC [103].

## 5. Impact of Physicochemical Attributes of Lipid Nanoparticles in Improving In Vivo Performance of Cancer Theranostics

A recent study conducted by Tahmasbi and associates established that physicochemical facade, predominantly shape and size, greatly influences the efficiency of the lipidic theranostic nanomedicines [104]. Mounting literature confirmed that spherical nanomedicines having a diameter within 20−100 nm lead to optimal tumor accretion owing to the EPR effect. However, dissimilar EPR attributes have been testified owing to nonspherical nanostructures (i.e., nanorods) [104,105].

The EPR effect refers to the selective buildup of lipidic theranostic nanomedicine at the tumor locations through extravasation via endothelial cells from the dripping vasculature. Accounting to the recent evidence, the physicochemical attributes of the lipidic theranostic nanomedicine comprising size, shape, charge, etc., possess greater potential in dictating tumor accumulation in contrast to active targeting on the exterior of nanomedicines [105]. Consequently, lipidic theranostic nanomedicine can be formulated for targeted tissues as well as for non-specific cell absorption by optimizing their physicochemical properties in the absence of targeted ligands [105]. Lately, it has been verified that shape greatly impacts cellular uptake [106,107]. Myriads of studies confirmed the diversified morphology-dependent anticancer efficacies for the same chemical compositions [108,109]. For the discoidal shape of lipidic nanomedicine having sizes <50 nm, tumor accumulation efficiency is not explored until recently [110,111,112,113]. For lipidic theranostic nanomedicine greater than 100 nm, needle-like rods have shown the maximum cellular uptake, trailed by shapes such as spheres, cylinders, and cubes, as evident in supporting studies [104,105]. For sub-100 nm lipidic theranostic nanomedicine, spheres demonstrated enhanced uptake by tumor cells over rods [104,105].

Nonetheless, the fluidity of lipid membranes can fluctuate with charge (negative or positive) and may persuade local gelation [104]. Neutral and slightly negative lipidic theranostic nanomedicine are found to maintain the longest half-life in blood [104,112,113]. Lipidic theranostic nanomedicine that has a positive charge could ensue the issues of platelet aggregation and hemolysis [104,112,113]. The competence of such a platform to fine-tune surface charge is another benefit in addition to size and shape adjustment that plays a crucial role in improvising biological behavior and clinical outcomes of lipid theranostic nanomedicines [104].

The absence of specificity with inactive targeting determined by the nanosize of lipidic theranostic nanomedicine has restricted efficiency. Mounting literature revealed that active targeting to the tumors might augment the intracellular uptake and lessen the lipidic theranostic nanomedicine’s spread in healthy tissues. A rational approach to attain this objective is to exploit specific interactions between the targeting molecules on the lipidic theranostic nanomedicine’s surface and overexpressed receptors of the cancer cells. Few ligands, namely transferrin, and folate can considerably enhance site-specific targeting [104]. Predominantly, folate has emerged as one of the targeting ligands for selective delivery of involved diagnostic and therapeutic agents owing to the overexpressed folate receptor (FR) in a myriad of tumor tissues, including kidney, lung, ovarian, cervical, breast, epithelial, brain and colon tumors, whereas limited in healthy organs and tissues. Folic acid being nonimmunogenic, unchanging over wide ranges of temperatures and pH values, can bind to the folate receptor after pairing with drugs or imaging markers. Consequently, folic acid has been extensively used as targeted therapy in cancers on account of its high binding affinity to FR, simplicity of conjugation to different lipidic theranostic nanomedicine, and the widespread distribution of FR in a substantial portion of human cancers [104]

## 6. Limitation of Lipid Nanoparticles-Based Cancer Theranostics

The major limitation with lipidic nanoparticles is their tendency to fuse, especially if the size of prepared nanoformulation is below 100 nm [114]. The fusion, in turn, results in increased non-uniformity and dispersity in size and escape of encapsulated contents from the lipid vesicles [114]. However, this issue can be addressed by covering the surface of lipid nanoparticles with polyethylene glycol coating. Another issue of great concern with lipidic nanoparticles is their stability over a longer duration of periods. It is certain that after storage for long periods, apparently intact lipid nanomaterials show a different biodistribution due to changes in physicochemical characteristics and alterations of the surface coating attributes [114].

Nonetheless, Carregal-Romero and associates have raised forth this very significant aspect of the influence of long-term stability of polyethylene glycol coating on contrast agent encapsulated nanoparticles and its in vivo fate [114]. They investigated the biodistribution of iron oxide nanoparticles, which were employed as dual contrast agents for MRI and SPECT imaging [114,115]. They developed these theranostic nanoparticles by co-precipitation of ^111^In-doped magnetic nanoparticles, followed by coating with polyethylene glycol [115]. Then they examined the physicochemical characteristics of freshly prepared nanoparticle solution and an aged nanoprobe solution that was stored for 9 months. The characteristic evaluation demonstrated comparable results of size distributions, zeta potentials, and morphology. However, after systemic administration of these two nanoparticle preparation in mice, completely distinct biodistribution pattern were observed [115]. The freshly prepared nanoprobe solution was mostly internalized in the kidney, whereas the aged nanoparticles were heavily concentrated in the liver. Therefore, the study outcomes concluded that there might occur some small level structural changes in polyethylene glycol coating that cannot be detected by dynamic light scattering and transmission electron microscopy, which have led to a remarkable alteration in in vivo behavior. Therefore this report established a substantial role of long-term stability polyethylene coating in dictating the biological behavior of theranostic nanoparticles [115].

The size of theranostic nanomedicine is also very crucial in determining its in vivo fate and clinical outcomes. It has been established in different studies that if the diameter of nanoparticles is larger than 100 nm then the particles get accumulated in the liver and spleen, whereas nanoparticles having a diameter smaller than 10–15 nm are usually eliminated by renal clearance [114]. Therefore nanomedicines with desirable particle diameters between 10 and 100 nm are supposed to have longer blood circulation times and accessibility to tumoral tissues and organs [114]. Furthermore, low polydispersity index (PDI) and size stability are very significant as they avert aggregation of nanoparticles, which could occur because of an inappropriate surface coating. The aggregated nanoparticle can be easily engulfed by the RES [114,115]. Nonetheless, polyethylene glycol coating is very effective in ensuring stability to the nanoparticles and thereby mitigating their opsonization, macrophage uptake, and RES clearance and increasing the blood circulation time [114].

Importantly, with every approved lipid nanomedicine, a possibility of intratumoral heterogeneity and variability of response to chemotherapy is evident. Unquestionably, the tumor microenvironment contributes largely in dictating how chemotherapeutic agents interact with cancer cells in that particular microenvironment, which in turn can impact proliferation, differentiation, morphology, and a range of cellular functions [116]. To envisage the clinical outcome of lipid-based theranostic nanomedicine, substantial emphasis should be given to universal heterogeneity issues with extraordinary conformity in terms of cancer cells, tumor microenvironment, and pathophysiological architecture [116]. However, with current theranostic and imaging modalities, complete biological approximation of the interaction of native tumor and a chemotherapeutic agent is not possible, which greatly hampers the accurate mapping and clinical findings.

## 7. Challenges in Clinical Translation of Lipid Nanoparticles for Cancer Theranostics

An extensive scope has been conveyed by lipidic theranostic nanomedicine in enhancing the health of humans through providing an understanding of diagnosis, prevention, and treatment of diseases. Even after substantial technological advancement in this area (as presented in Table 2), there is still a long way ahead for lipidic cancer nanotheransotic in becoming a new criterion for the diagnosis and treatment of diseases. Nano-bio interaction is a prime opposition for the transferal of lipidic theranostic nanomedicine to clinics. Disorders like inflammation, immunoreactions, or related illness can come up when a contrasting agent in lipidic theranostic nanomedicine interacts with biological material because of its incompatibility or potential toxicity. The extent of toxicity is immensely based on some parameters, which include the solubility of the nanoformulation, size, and zeta-potential [117]. Once entered into a biological system, nanovesicles tend to interact with proteins, and that results in the development of ‘corona’ on its surface. Such adsorption, in turn, results in the alteration of their stability, biodistribution, dispersibility, toxicity profile, pharmacokinetics, and size [118,119]. This has also been shown in many studies that adverse immunogenic reactions and allergies are happening by nanoparticles [120,121]. Hence, it becomes vital to study the physicochemical characteristics of nanomedicines for heterogeneity and pathophysiology of human diseases. More importantly, a generalized size outlook of lipidic theranostic nanomedicine is not possible as chemotherapy is distinct for every patient, and this may pose a hurdle in clinical translation [122]. Nanomedicines with good therapeutic efficacy might not be a good diagnostic tool necessarily, as suggested by the evidence. In a recent study, it was found that tumor uptake and tumor visualization performed with anti-EGFR coated gold nanoparticles with 20 nm size showed increased tumor uptake, whereas the same gold nanoparticles of 50 nm size illustrated excellent CT contrast [123]. This study suggested that the size-dependent distribution of theranostic nanomedicines in tumors limits its use as a theranostic agent [123]. Hence, the safety profile of nanotheransotic in humans continues to be a major concern for which long-term monitoring of patients in both early and advanced phases of clinical trials is required. One more important obstruction for the clinical translation of lipidic theranostic nanomedicines is the complexity in formulating a reproducible and controllable synthesis process. Nanomedicines synthesis on a large scale faces challenges like varied physical and chemical characteristics, low yield, and insufficient batch-to-batch reproducibility. The complex and laborious manufacturing process of nanomedicines makes it difficult to focus on physic-chemical attributes as the emphasis is more on quality and cost [122,124]. It becomes a task to produce lipidic theranostic nanomedicines on a large scale as they constitute more precise chemistry and multifunctional unit. Moreover, control along with good manufacturing practices are required to boost lipid theranostic medicine’s reach to clinics from a laboratory.

Another important issue that is required to be addressed is the extensive gap between regulatory authorities and the scientific community. Many government agencies are monitoring the commercialization of nanomedicine based on regulatory issues related to the safety profile, quality control, patent protection, and manufacturing practices. Lack of clear regulatory and safety guidelines affects the timely and effective translation of lipidic theranostic to market [125,126]. Even though the general regulatory standards have been cleared by the nanomedicines presently available in the market, further revision is required to be sure of the safety, efficacy, and quality of other nano theranostic for human use since the present standards might not be sufficient.

### Approach to Overcome the Challenges

A lot of research efforts are required to overcome the biological barriers associated with lipidic cancer nanotheransotic. It is important to have a profound understanding of the correlation of disease heterogeneity and patient biology with nanomedicine, which is also the prime reason for the failures of promising nanoformulation in clinical trials. One of the strategies for mitigating clinical translation failures is the arduous assessment of nanoformulation in various animal models before starting the clinical trials. Expedient information regarding the suitability of lipidic theranostic nanomedicine can be obtained through preclinical studies before treatment and imaging of human subjects [127]. Nanotoxicology profiles consisting of standardized assay protocols for immunotoxicity, genotoxicity, and cytotoxicity should be to be implemented and followed to evaluate the potential risk in patients [128].

Academic laboratories are coming up with nanomedicine-based drug-delivery systems with great emphasis on new technological and scientific developments that succeed at a small scale. These laboratories normally know the technical issues, which occur in the industry for the commercializing processes. A strong collaboration among pharmaceutical companies and academic laboratory groups is required to be established to bridge this gap. There is a need to develop modified rules, which will be listed under good manufacturing practices that are suitable for large-scale synthesis of lipidic theranostic nanomedicines. To identify the key process and formulation variables for nanomedicine optimization and address batch-to-batch variation, optimization software such as Aspen (AspenTech, Bedford, MA, USA) can be implemented in an industrial setting. For tightly controlled and robust manufacturing, this might be instrumental [129]. In conclusion, a positive outcome in manufacturing depends highly on how well the personnel is trained regarding the challenges, hurdles, and specificities related to the products. There is no doubt regarding the influence of lipidic theranostic nanomedicines on the health of humans in the clinic still. If the aforementioned lessons are applied in the early stages of development, it can help the producers prepare to develop efficient products.

## 8. Conclusions

The present manuscript brings forth the latest research updates in the field of lipid-based nanocarriers on cancer theranostic. The study findings are very motivating and strongly encourage a splendid exploration of lipidic nanocarrier in the area of cancer theranostic and make the outcomes clinically swappable. Moreover, this review provides a sound discussion over the impact of physicochemical attributes of lipid nanoparticles in improving in vivo performance of cancer theranostics. The review also discusses the limitations and suggests relevant solutions for the successful development of lipid-based cancer theranostic nanomedicines of improved attributes to pave their way to the clinics. Additionally, this review will provide a convenient guide for the researcher to know the significant findings of the recent studies carried out in the field of lipid nano carrier-based cancer theranostic in the last few years.

## Figures and Tables

**Table 1:** Theranostic application of lipidic nanomedicines for cancer therapy.

Lipidic Nanocarrier	Chemotherapeutic Agent	Diagnostic Agent/Modality	Experimental Model	Theranostic Outcome	Ref.
Nanoemulsion	PDT	fluorinated cryptophane-A and porphyrin self-assembled onto the surface of fluorinated nanoemulsions-^19^F MRI and fluorescence imaging	Xenograft A549 tumor mice.	A high therapeutic efficacy; low toxicity; high tumor accumulation of nanoemulsion	[38]
	PDT	Fluorescence probe/photoacoustic/^19^F magnetic resonance multimodal	A375 melanoma xenograft model	The remarkable efficiency of PDT on hypoxic solid tumors via a single injection of the drug; outstanding diagnostic ability	[39]
	Doxorubicin and Paclitaxel	Perfluorohexane (PFH) vaporized bubbles as an Ultrasound contrast agent	MCF-7 cells	Markedly enhanced PFH-NEs targeting and lodging in tumor region with simultaneous treatment monitoring.	[40]
	Paclitaxel and PDT	Porphyrin NE shell-based photoacoustic imaging and fluorescence imaging; CT contrast	Mice bearing tumors	multimodal cancer imaging, highly efficient phototherapy and image-guided drug delivery	[41]
Liposomes	Doxorubicin HCl	gold nanoparticles (AuNPs) and emissive graphene quantum dots (GQDs)	Breast tumor-bearing mice models	specific and enhanced cellular uptake, prolonged internalization in tumor and substantial contrasting and therapeutic efficacy	[49]
	Paclitaxel and vinorelbine	Tc-99m radiolabeled	NSCLC tumor-bearing C57BL/6 mice	Effectively inhibited tumor growth completely restricted lung metastasis	[50]
	Gefitinib and simvastatin	Fluorescence imaging	Brain Metastasis (BM) mouse model developed by intracranial transplant of the H1975 NSCLC cells	Efficient permeation across the blood–brain barrier and high capability of reversing drug resistance.	[51]
	Doxorubicin	Acoustic cluster therapy (ACT); Ultrasound insonition	orthotopic human tumor xenografts in athymic mice	Substantial increase therapeutic efficacy of Doxil^®^ when combined with ACT	[52]
	Paclitaxel and ultrasound responsive drug delivery	Ultrasound imaging	MiaPaCa-2, Panc-1, MDA-MB-231, and AW-8507 cell lines	300-fold higher anticancer activity in contrast to ABRAXANE.	[53]
SLN	Paclitaxel and siRNA	Quantum dots	A549 cancer cells	Efficient in situ visualization of intracellular translocation of SLNs into cancer cells.	[54,62]
		^64^Cu, PET imaging, and ex vivo gamma counting	Mice	^64^Cu-radiolabelled SLN and their biodistribution was efficiently quantitatively evaluated	[59]
NLC	Paclitaxel	Quantum dots	HepG2 cells/Female Kunming mice	Imaging established splendid capability of the co-loaded NLC to specifically target and detect the H22 tumor.	[65]
	IR 780 and Photothermal therapy	fluorescent probe coumarin 6	4T1-luc cell line in BALB/c female mice	Notably enhanced photothermal anti-tumor effect as well as anti-metastatic efficacy in vivo	[64]
	Camptothecin	Quantum dots	Melanoma cells	camptothecin accumulation in melanomas increased by 6.4-fold	[66]
	Paclitaxel	^99m^Tc(CO)^3+^	Wistar Albino rats.	Substantially high cellular uptake and concurrent imaging	[67]
Lipid nanocapsule	Celecoxib and honokiol	fluorescent mercaptopropionic acid-capped cadmium telluride was coupled with quantum dots as an imaging probe	human breast cancer cells: MCF-7 and MDA-MB-231; EAT model	Highly improved and superior anticancer efficacy; Efficiently traceable LNC internalization	[69]
Lipid-Polymer Hybrid	Platinum (IV) (Pt(IV)) prodrug	(glutathione (GSH)-sensitive platinum (IV) for Ultrasound imaging	αvβ3- and αvβ5-positive SKOV3 human ovarian tumor cells and αvβ3- and αvβ5-negative A2780 human ovarian tumor cells	Significant therapeutic efficacy and limited side effect	[71]

**Table 2:** Lipidic nanocarrier based cancer theranostic in clinical stage of progress.

Lipidic Nanocarrier	Attributes	Cancer Type	Sponsors	Clinical Trial ID/Phase
Liposomes	Evaluating Immunogenic Chemotherapy Combined With Ipilimumab and Nivolumab in Patients With Metastatic Luminal B Breast Cancer	Breast Cancer	Oslo University Hospital	NCT03409198, Phase 2B
Liposomes	To study the distribution profile and radiation dosimetry of 188Re-BMEDAliposomes.	Tumors	Nuclear Energy Research Institute of Taiwan.	NCT02271516 Phase 1
Liposomes	To study the MTD of EphA2 siRNA –encapsulated liposomes, evaluate efficacy in the tumor cell, which we cannot be cured by treatment.	Solid Tumors	M.D. Anderson Cancer Center National Cancer Institute (NCI)	NCT02191878 Phase 3
Lipid-based Nanoparticles	To study proposes targeted delivery cytotoxic drugs, via formulated LTSL activated by using focused ultrasound (FUS).	Liver Tumor	University of Oxford	NCT02181075 Phase 1

## Data Availability

Not applicable.
